# An Alternative to Current Therapies of Functional Dyspepsia: Self-Administrated Transcutaneous Electroacupuncture Improves Dyspeptic Symptoms

**DOI:** 10.1155/2014/832523

**Published:** 2014-10-30

**Authors:** Ting Ji, Xueliang Li, Lin Lin, Liuqin Jiang, Meifeng Wang, Xiaopin Zhou, Ranran Zhang, Jiande DZ Chen

**Affiliations:** ^1^Division of Gastroenterology, Department of Gastroenterology, First Affiliated Hospital, Nanjing Medical University, Nanjing 210029, China; ^2^Division of Gastroenterology and Hepatology, Johns Hopkins Medicine, Baltimore, MD 21224, USA

## Abstract

Functional dyspepsia is of high prevalence with little treatment options. The aim of this study was to develop a new treatment method using self-management transcutaneous electroacupuncture (TEA) for functional dyspepsia (FD). Twenty-eight patients with FD were enrolled and underwent a crossover clinical trial with 2-week TEA at ST36 and PC6 and 2-week sham-TEA at nonacupuncture sham-points. Questionnaires were used to assess symptoms of dyspepsia and quality of life. Physiological testing included gastric emptying and electrogastrography. It was found that (1) TEA but not sham-TEA significantly improved dyspeptic symptoms and 4 domains in quality of life; improvement was also noted in self-rated anxiety and depression scores; (2) gastric emptying was significantly and substantially increased with 2-week TEA but not sham-TEA; and (3) gastric accommodation was also improved with TEA but not sham-TEA, reflected as increased ingested nutrient volumes at the levels of satiety and maximum tolerance. These findings suggest a therapeutic potential of self-administrated TEA method for functional dyspepsia, possibly attributed to improvement in gastric motility.

## 1. Introduction

According to the Rome III criteria [1], functional dyspepsia (FD) is defined as a symptom originated from gastroduodenum, characterized by upper abdominal pain, burning, postprandial fullness, and early satiety. The prevalence of FD was reported to be about 25% of the general population [2]. The main pathophysiological mechanisms of FD include increased visceral sensitivity, impaired gastric accommodation, and delayed gastric emptying.

Acupuncture is a Traditional Chinese Medicine treatment which has been applied for thousands of years for a variety of diseases, including gastrointestinal diseases [3–5]. In the last three decades, acupuncture has been gradually accepted by physicians and patients in modern medicine. The most commonly used acupuncture points (acupoints) for the treatment of gastrointestinal symptoms are Zusanli (ST-36) and Neiguan (PC-6) [6]. Electroacupuncture (EA) at ST-36 and PC-6 has been documented to increase the regularity of gastric slow waves and accelerate gastric emptying of liquids in animals [7]. Transcutaneous electroacupuncture (TEA), which uses electrodes instead of needless, is proved as effective as traditional acupuncture [8].

A watch-size microstimulator, called Neuromodulation Regulator for Gastrointestinal Functions (SNM-FDC01, Ningbo Maida Medical Device Inc., Ningbo, China), has been developed and could be used for TEA in treating various illnesses. Using this device, the patient can wear it on the arm and/or leg and resume normal daily life; the treatment was completely noninvasive and could be delivered a few times daily instead of every week. Thus, it is expected to be more appropriate for chronic home-based treatment with increased efficacies. However, it was unknown which beneficial effects could be observed in patients with FD when TEA was used.

Our main purpose of this study was therefore to investigate the effects of TEA on dyspeptic symptoms and quality of life and possible mechanisms involving gastric slow waves and gastric emptying in patients with FD.

## 2. Materials and Methods

### 2.1. Subjects

We screened outpatients with functional dyspepsia who visited Gastroenterology Division of First Affiliated Hospital of Nanjing Medical University from August 2013 to April 2014. Inclusion criteria were (1) meeting the Rome III criteria for postprandial distress syndrome (PDS) of FD; (2) age 21–65 years; and (3) willingness to follow the treatment plan and sign the informed consent. Patients with one or more cases of the following were excluded: (1) taking drugs which would affect gastric motility, such as prokinetic drugs, anticholinergic drugs, and dopamine; (2) history of gastrointestinal surgery; (3) pregnancy or preparing to be pregnant; (4) diabetes mellitus; (5) allergic to skin preparation; (6) knowing acupuncture points. At last, 28 patients with FD were enrolled, including 15 males and 13 females, age 27–60 years with a mean age of 44.1 ± 9.4 years.

### 2.2. Experimental Protocol

A method of single-blind randomized crossover trial was adopted. Experimental subjects were randomly divided into two equal groups (groups A and B). The treatment protocol was similar to the one described previously [9]. In brief, in group A, patients were first treated with TEA for 2 weeks and a washout period of 1 week and then treated with sham-TEA for 2 weeks. Patients in group B were treated in a reversed order. At the enrollment, patients were trained to put electrodes on the two acupuncture points (ST-36 and PC-6) or two sham-points of their bodies.

Pulse trains were used for TEA with a train on-time of 2 seconds and off-time of 3 seconds, pulse width of 0.5 ms, pulse frequency of 25 Hz, and pulse amplitude ranging from 2 to 6 mA, depending on the tolerance of the subject. Sham-TEA stimulation was performed using the same method except that electrical stimulation was performed at sham points (nonacupoints): the sham-point for PC6 was about 15 cm up (to the elbow) and lateral to PC6 and the sham-point for ST36 was about 10 cm down (to the knee joint) and lateral to ST36. During the washout period, the patients received neither treatment of TEA or sham-TEA nor medications. TEA or sham-TEA was delivered using a match-size stimulator (SNM-FDC01, Ningbo Maida Medical Device, Inc. Ningbo, China) for 2 hrs after each meal (3 times daily). Patients came to the hospital 4 times during the study, at the beginning and end of each treatment.

### 2.3. Measurements

A number of measurements were made during each of the hospital visits, including questionnaire on dyspepsia symptoms, SF-36 quality of life questionnaire, the Zung self-rating anxiety/depression scale, the electrogastrography test, the gastric accommodation test, and gastric emptying test.

#### 2.3.1. Dyspepsia Symptom Scale

The patient was asked to complete a questionnaire including nine dyspeptic symptoms (upper abdominal pain, upper abdominal discomfort, postprandial fullness, upper abdominal swelling, early satiety, nausea, vomiting, and excessive belching and heartburn) and their severities (0–3: none-severe) and frequencies [10].

#### 2.3.2. Quality of Life Assessment (SF-36)

It included 36 questions describing 8 dimensions: physical functioning (PF), role limitations due to physical problems (RP), bodily pain (BP), general health perception (GH), vitality (VT), social functioning (SF), role limitations due to emotional problems (RE), and mental health (MH). Each dimension had a score range from 0 to 100; the higher the score, the better the quality of life [11].

#### 2.3.3. The Zung Self-Rating Anxiety/Depression Scale

Each of the self-rating anxiety scale (SAS) and self-rating depression scale (SDS) system included 20 questions about anxiety/depression. The higher the score was, the more anxious or depressed the patient was [12].

#### 2.3.4. Electrogastrography

The gastric slow waves were recorded using a 4-channel electrogastrogram (EGG) device (MEGG-04A, Ningbo Maida Medical Device Inc., Ningbo, Zhejiang, China). Six cutaneous electrodes were placed on the abdominal skin surface using a previously established method for the EGG recordings after a careful skin preparation [13]. The subject was lying in a bed and was asked to minimize movement and not to talk or read during the test. The EGG was recorded for 30 min in the fasting state and 60 min after a standard test meal composed 50 g of bread, 100 g of ham sausage, and 200 mL of water.

Previously validated spectral analysis software was applied to derive the following EGG parameter from the EGG recordings [14]: percentage of normal 2–4 cycles/min slow waves, representing the regularity of gastric slow waves.

#### 2.3.5. Nutrient Drink Test

The noninvasive nutrient drink test was used to assess gastric accommodation [15, 16]. The patient was fasted for at least 8 hours before the test. In the test, the patient was asked to drink at a speed of 30 mL/min; the drink was composed of 50 g of Cola Cao (chocolate flavor) and 100 g of Nestle full cream milk powder, dissolved in 840 mL of water (carbohydrates: 48%, fat: 39%, protein: 13%, and energy density: 1.0 Cal/mL). The volume for the feeling of satiety and the volume for the maximum tolerance were used to assess gastric accommodation.

#### 2.3.6. Gastric Emptying

After at least 8 hour fasting, the patient was asked to consume a standard meal (instant noodles 80 g, ham sausage 50 g) mixed with 20 small strips of 10 mm radiopaque bariums [17]. Five hours later, an abdominal radiogram was made, the number of barium markers in the stomach was counted, and the rate of gastric emptying was determined.

### 2.4. Statistical Analysis

Data are presented as mean ± SE. Analysis of variance (ANOVA) was used for 2 × 2 crossover model (Stata/SE 12.0 for windows, Stata Corp LP., College Station, TX, USA).* Levene's* test was used for equality of variances.* SNK's* test was used for multiple comparison (SPSS 19.0 for windows, SPSS Inc., Chicago, USA). Statistical significance was assigned for *P* < 0.05.

## 3. Results

### 3.1. Effects of TEA on Dyspeptic Symptoms

**Figure 1:**
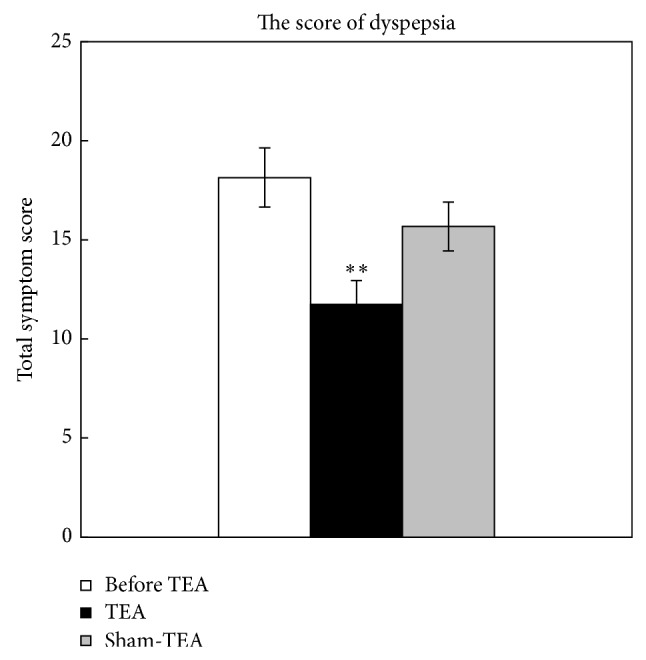
Effects of TEA on dyspeptic symptoms. ^**^ *P* < 0.01, versus before TEA.

TEA treatment was effective in improving dyspeptic symptoms. There was no sequence effect (*P* = 0.0791) on this trial but there was a period effect (*P* = 0.0300) (Table 1). The multiple comparisons revealed the following (Figure 1): there was a significant difference between before and after the TEA treatment in the symptom score (18.14 ± 1.49 versus 11.75 ± 1.19, *P* = 0.004); however, there was no significant difference between before and after the sham-TEA treatment in the symptom score.

### 3.2. Effects of TEA on Quality of Life

**Figure 2:**
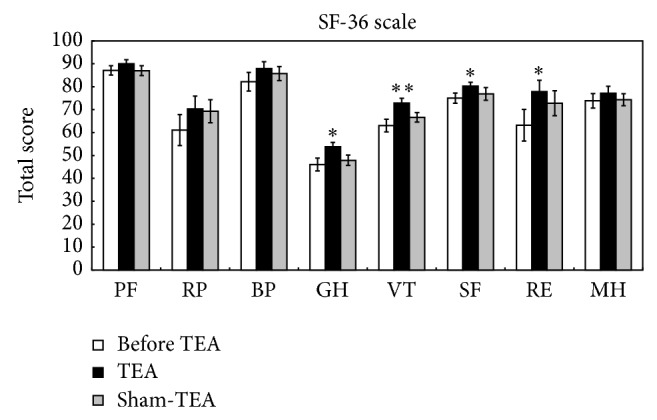
Effects of TEA on quality of life. ^*^ *P* < 0.01, versus before TEA; ^**^ *P* < 0.01, versus before TEA.

A significant treatment effect was noted in 4 dimensions of the SF-36 (*P* = 0.0217, GH; *P* = 0.0007, VT; *P* = 0.0040, SF; *P* = 0.0424, RE) but not in other 4 dimensions (*P* = 0.0803, PF; *P* = 0.2007, RP; *P* = 0.3035, BP; *P* = 0.1455, MH). As shown in Figure 2, there were significant differences between before and after TEA treatment in GH (46.04 ± 2.83 versus 53.79 ± 1.90, *P* = 0.032), VT (63.04 ± 2.81 versus 72.86 ± 2.12, *P* = 0.008), SF (75.00 ± 2.19 versus 80.27 ± 1.69, *P* = 0.039), and RE (63.18 ± 6.94 versus 77.89 ± 4.95, *P* = 0.029). In the other hand, no significant difference was found between before and after the sham-TEA treatment in these dimensions (GH, VT, SF, and RE).

### 3.3. Effects of TEA on Anxiety and Depression

**Figure 3:**
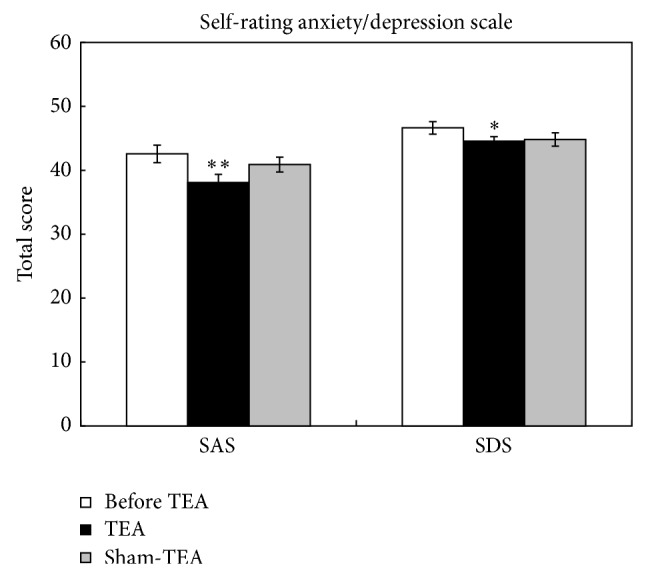
Effects of TEA on self-rated anxiety and depression. ^*^ *P* < 0.05, versus before TEA; ^**^ *P* < 0.01, versus before TEA.

A significant difference was found between before and after TEA treatment for both SAS and SDS (*P* = 0.0022 and 0.0422, resp.). It showed that after TEA treatment, both SAS and SDS scores were lower than before (Figure 3). Accordingly, one-way ANOVA was used to investigate the treatment effects. A significant difference was noted between before and after the TEA treatment in both SAS (42.57 ± 1.37 versus 38.11 ± 1.24, *P* = 0.006) and SDS (46.64 ± 0.99 versus 44.54 ± 0.70, *P* = 0.037). No such differencewas noted with the treatment of sham-TEA.

### 3.4. Effects of TEA on Normal Gastric Slow Waves

**Figure 4:**
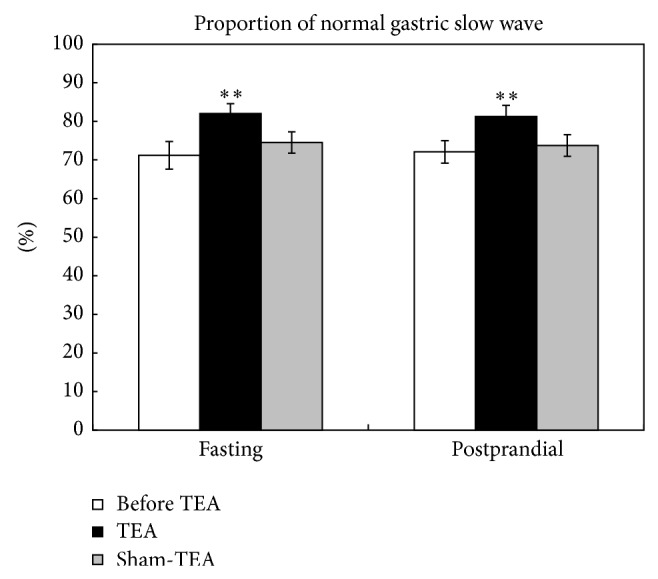
Effects of TEA on the percentage of normal gastric slow waves. ^**^ *P* < 0.01, versus before TEA.

A significant difference was found between before and after TEA treatment in the percentage of normal gastric slow waves in both fasting and fed states (*P* = 0.0032 and 0.0001, resp.). It showed that after TEA treatment, the percentages of normal gastric slow waves in both fasting and fed states were higher than before. Then one-way ANOVA and multiple comparisons were performed for further authentication (Figure 4). In the effect of TEA on fasting slow waves, there was a significant difference between before TEA and TEA (71.2 ± 3.6% versus 82.0 ± 2.5%, *P* = 0.005). Significant difference was also found between before TEA and TEA (72.0 ± 2.9% versus 81.3 ± 2.9%, *P* = 0.008) on postprandial slow waves. However, sham-TEA did not show any significant effect on gastric slow waves.

### 3.5. Effects of TEA on Gastric Accommodation

**Figure 5:**
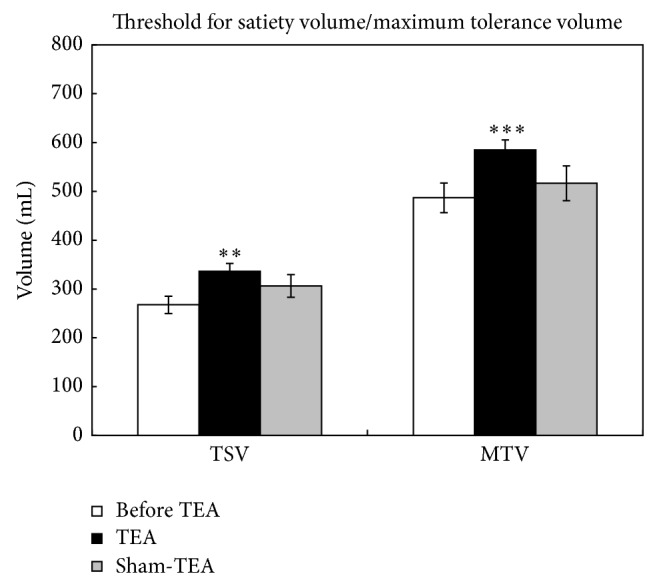
Effects of TEA on gastric accommodation. TSV: threshold for satiety volume; MTV: maximum tolerance volume. ^**^ *P* < 0.01, versus before TEA; ^***^ *P* < 0.001, versus before TEA.

There were significant differences between treatment groups in both ingested volumes for satiety and maximum tolerance (*P* = 0.0027, 0.0008, resp.) (Figure 5). Treatment group meant the patients who had took TEA treatment. Multiple comparisons showed a significant increase after the TEA treatment in both the ingested volume for satiety (267.5 ± 17.9 mL versus 336.6 ± 15.8 mL, *P* = 0.008) and the maximum tolerable volume (486.8 ± 30.3 mL versus 585.0 ± 20.4 mL, *P* < 0.001). The treatment of sham-TEA, however, did not yield such an increase.

### 3.6. Effects of TEA on Gastric Emptying Rate

**Figure 6:**
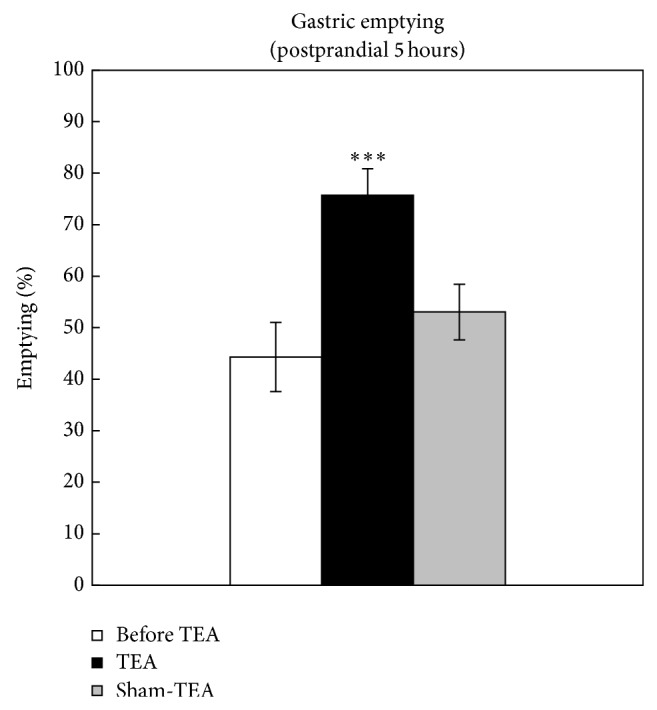
TEA accelerated gastric emptying in FD patients. ^***^ *P* < 0.001, versus before TEA.

The difference between the treatment groups was statistically significant (*P* = 0.0017). The multiple comparison analysis revealed a significant increase after the TEA treatment in the rate of gastric emptying (44.3 ± 6.7% versus 75.7 ± 5.1%, *P* < 0.001) (Figure 6). However, such an increase was not noted with the sham-TEA treatment.

## 4. Discussion

Currently FD is mainly being treated with prokinetic and antacid drugs [18]. However, a considerable number of FD patients respond poorly to medical therapies. In this study, we used TEA to treat patients with FD and found significant improvement in dyspeptic symptoms, anxiety and depression, and quality of life. Physiological measurements revealed improvement in gastric motility, reflected as improvement in gastric emptying and increase in regularity of gastric slow waves.

In previous studies, we conducted investigation on acupuncture and found that acupuncture at ST-36 and PC-6 was helpful in improving gastrointestinal motility functions [19]. However, traditional acupuncture involves the use of needles; moreover, the treatment has to be carried out in hospital. Contrarily, the proposed method of TEA had advantages of being noninvasive and self-administrative. In this study, patients who were treated with TEA had good compliance with zero dropouts. That is, the proposed TEA method is expected to be well received by patients.

A significant decrease in dyspepsia symptom score was noted with TEA but not sham-TEA in this study. This represented a true therapeutic effect. Some previous studies reported similar effects on symptoms associated with gastric dysmotility using acupuncture or electroacupuncture [20, 21]. Similar symptom improvement was found in a previous TEA study using a TENS unit [9]; however, the FD patients were not subclassified into PDS and EPS. In the present study, only patients with PDS were enrolled in the study.

The SF-36 scale, reflecting the physical function and mental health, had been widely used in clinical evaluation of patients' quality of life in the past decades [22–25]. It has been well established that the target of FD management should not only be alleviating physical symptoms but also be improving the quality of life. As a result, not only biomedical indicators but also assessment of the quality of life was necessary to reflect the health of patients; the latter should be the ultimate goal of an effective treatment. Studies had shown that quality of life for most FD patients was lower than healthy people. The results of this study showed TEA was effective on four dimensions (GH, VT, SF, and RE) of the SF-36, demonstrating that TEA is effective in improving the patients' quality of life.

In long-term studies, researchers had realized the important pathophysiological mechanisms involving psychological and social factors in patients with FD. The common mental disorders in FD patients involve mostly anxiety, depression, and somatization disorders. One previous study pointed out that FD patients had a higher incidence in anxiety/depression than those with organic dyspepsia [26]. A study in 2006 reported improvement with electroacupuncture in anxiety symptoms in patients with FD [27]. In the current study, a significant decrease was observed with TEA in both scores of anxiety and depression. To the best of our knowledge, this was the first study demonstrating simultaneous improvement in both psychological factors and gastric motility. These combined ameliorating effects make the TEA attractive for the treatment of FD.

About 70 of FD patients were reported to have impaired gastric slow waves or abnormal EGG [28]. It is noteworthy to mention that a previous study of TEA at ST-36 and PC-6 using a TENS unit was shown ineffective in improving gastric slow waves measured by EGG [9]. It was suggested that TEA might not treat disorders induced by gastric myoelectrical disturbances and that the lack of efficacy in normalizing gastric slow waves could be attributed to the indirect stimulation of acupuncture points without insertion of needles. Different from the previous study, our current results showed significant and substantial improvement in gastric slow waves in both fasting and postprandial states. The improved performance of the TEA in this study could be attributed to the increased capacities of the newly developed watch-size stimulator. This new stimulator was capable of delivering a wide range of stimuli, such as increased pulse width (0.5 ms was used in this study whereas the TENS unit is limited to 0.3 ms). In addition, the patients in this study resumed regular daily life during the treatment, whereas the patients in the previous study were restrained in bed.

Reduced gastric accommodation is known to cause symptoms of early satiety and bloating. Using the noninvasive nutrient drink test [29], we have shown that TEA was able to improve gastric accommodation in FD patients. The ingested volumes for both satiety and maximum tolerance were significantly increased after the TEA treatment. This was a novel finding. The improvement in gastric accommodation concurrent with the improvement in gastric emptying is of great clinical significance because a compromise must be made in medical therapies: muscle relaxants are needed to improve gastric accommodation but this would delay gastric emptying; on the other hand, prokinetics are required to accelerate gastric emptying but this would further reduce gastric accommodation.

The gold standard for measuring gastric emptying is scintigraphy, either with digestible solids or liquids. Unfortunately, this method is expensive and of limited availability. Stutter pointed out that the result of using radiopaque markers to determine solid gastric emptying was close to scintigraphy and the use of radiopaque markers is simple and reliable [30]. Previously, manual acupuncture at the ST-36 and PC-6 points was reported to accelerate solid gastric emptying in FD patients with delayed gastric emptying and relieves dyspeptic symptoms in FD patients with normal gastric emptying [27]; electroacupuncture at the same points improved gastric emptying in animals [7]. In the present study, the needleless TEA significantly and substantially accelerated gastric emptying in FD patients. Based on these findings, we could infer that chronic needleless TEA is as effective as EA or acupuncture in improving gastric emptying of solids.

In conclusion, the proposed needleless self-administrated method of TEA is effective in treating dyspeptic symptoms and improving quality of life and psychological state. These ameliorating effects could be attributed to the improvement in gastric emptying and slow waves. This noninvasive method of TEA could be considered as an alternative for treating patients with functional dyspepsia.

## Figures and Tables

**Table 1:** Sequence, treatment, and period effects on symptom scores.

Source of variation	Partial SS	Df	MS	*F*	Prob > *F*
Sequence effect	60.07	1	60.07	3.34	0.0791
Treatment effect	0.00	1	0.00	0.00	0.0040
Period effect	216.07	1	216.07	29.89	0.0300

Total	931.71	55			

In improving symptom score, treatment effect was significant (*P* = 0.0040). There was no sequence effect (*P* = 0.0791), but some period effect existed (*P* = 0.0300).
